# Hospital Meetings, &c.

**Published:** 1901-05-11

**Authors:** 


### HOSPITAL MEETINGS, &c.

### ROYAL HOSPITAL FOR DISEASES OF THE CHEST.

The festival dinner of the Royal Hospital for Diseases of the Chest, City Road, was held on Monday, 6th instant, at the Hôtel Métropole, Sir Andros de la Rue, the chairman of the Council, presiding.

After the customary loyal toasts had been duly honoured, the Chairman proposed "Success to the Hospital." He said: Gentlemen,—I rise to propose what is termed the toast of the evening, "Success to the Royal Hospital for Diseases of the Chest," a toast sure to be well received, because, by your kind presence here this evening, you give practical proof of the great interest you take in the institution. I do not intend to inflict upon you a long account of the growth of the hospital, but I would remind you that it was founded by the Duke of Kent in 1814; and, also, that it was the first hospital in Europe for the study and treatment of consumption. It started on a very small scale, but was so much appreciated and sought after by the poor, suffering from pulmonary disease, that its work grew year by year until 1883, when the number of patients seeking advice was such that the accommodation was found to be quite inadequate. It was therefore decided to appeal for funds to provide the necessary accommodation. His Majesty the King was graciously pleased, as Prince of Wales, to preside at a dinner in that year, which was highly successful, and resulted in the building of the present hospital, in which about 800 in-patients are treated yearly. The attendance of out-patients exceeds 26,000 annually. A special feature in connection with out-patients is that all necessary medicines are provided gratuitously. I regret to say that the reliable income of the hospital from annual subscriptions is less than one-third of the expenditure, and therefore you will realise that, from time to time, we get into troublous waters. It then becomes necessary to appeal for ballast, in the shape of donations, to enable us to steer the good ship into smooth water again. Subscriptions have, unfortunately, not come in as freely as usual during the last year or two, no doubt owing to the exceptional claims made upon the public by the War Relief Funds and by the Indian Famine Fund. Our financial position is therefore, at the moment, not so rosy as it might be; and, to add to our difficulties, the Prince of Wales's Hospital Fund Committee and our own medical staff insist upon the imperative necessity of our providing a suitable home for our nurses. To acquire a freehold site, build and furnish a Nurses' Home, will cost some £7,000, to which must be added £3,000 required for the ordinary work of the hospital. I therefore appeal to you for £10,000 ballast. The subscriptions will show that a good portion of this has been already given. I now earnestly appeal to you, Gentlemen, to open your hearts and your purse-strings, and provide the required balance. It would be sad for our ship to arrive in port with any "list" on her. I hope that by the end of the evening the secretary will be able to announce that your donation list, which he will shortly collect, has trimmed our good ship the "Royal Hospital." The toast of the evening is, "Success to the Hospital." Now, I feel that success depends upon two factors:—Firstly, liberal financial support on the part of the charitable public; secondly, efficient management of the hospital. It appears to me that the first is dependent upon the second; for the charitable will clearly only freely give to an institution which they know to be well managed. I will, therefore, as chairman of this dinner, say a few words upon the management of the Hospital for Diseases of the Chest. I am in a good position to do so, as no one knows the chairman of the Council of Management better than I do, and I may say that he hides nothing from me. If you will look over the list of the Council of Management, you will see names in the forefront of the commercial world—such names as Lord Rothschild, Mr. Hope Morley, Colonel Makins, Mr. Gilliat, Mr. Glyn, and many others—and I submit that the fact of such well-known men devoting their time to the management of the hospital should be a sufficient guarantee to the charitable that their money is well spent. I myself have been a member of the Council for 25 years, and have had the honour of being chairman of the Council for the past 10 years, so I am in a position to testify to the harmonious and efficient working of the hospital. To work with such a Council is a genuine pleasure, which is enhanced by the cordial and more than friendly co-operation of the medical staff. Our medical staff—I must stop, for I see Colonel Makins looking reproachfully at me, and I have just received a hint from the secretary that it does not devolve upon me to speak upon this subject; so I must refrain from expressing my heartfelt appreciation of their work, my gratitude to them, and from saying what I feel. The feeling of the Council will be much more eloquently and ably expressed, later on, by my friend Colonel Makins, who himself has been a member of the Council for nearly thirty years. Hospital work deals with the suffering side of humanity and is of necessity somewhat sad; but, oddly enough, consumptive patients are almost always hopeful. I remember, some years ago, visiting an in-patient—one of our own workpeople—who, the doctor told me, could not live 48 hours. He expressed his heartfelt thanks for the great kindness he had received in the hospital, and said that he felt so much better that he hoped to return to work the following week. Poor fellow, he died the next morning. I merely instance this to point out that, even when the hospital treatment fails to save life, it makes the last few weeks of life happier, and the inevitable end less sad. That the hospital is beloved by the suffering poor is well known to those who work among them. In conclusion, gentlemen, I earnestly appeal to you to assist in carrying on the great work of the Royal Hospital for Diseases of the Chest, and I would ask you to remember that, even if your support does not in all cases save life, it at any rate alleviates suffering; and, therefore, I confidently propose the toast of the evening, "Success to the Royal Hospital for Diseases of the Chest," and may it long continue to carry on its good work.

The toast was drunk with enthusiasm, after which the secretary read the list of subscriptions received. The total amount, £5,809, was, he said, the largest by far ever compiled on behalf of the hospital, the previous best total, £4,730, having been in 1883, when the Prince of Wales presided. Other notable amounts were, in 1893, £3,187, when Lord Rothschild took the chair; in 1896, £3.479, when Lord Charles Bruce presided; and £4,630 in 1898 under their treasurer, Mr. Hope Morley.

The other toasts were "The President, Lord Rothschild," proposed by the Hon. Claude Hay, M.P.; "The Chairman and Council;" and "The Medical Staff and other Honorary Officers."

